# The Fate and Degradation of Genetically Modified Soybean DNA Along the Soybean Oil Processing Chain: Quantitative Tracking and Comparison of Degumming Methods

**DOI:** 10.3390/foods15203718

**Published:** 2026-10-19

**Authors:** Yimiao Xia, Mengru Guo, Shengwen Cui, Boye Liu, Yu Chen, Fusheng Chen

**Affiliations:** 1 College of Food Science and Engineering, Henan University of Technology, Zhengzhou 450001, China; xiayimiao@haut.edu.cn (Y.X.); gmr2455990731@163.com (M.G.); liuboye@haut.edu.cn (B.L.); chenyu@haut.edu.cn (Y.C.); 2 National Engineering Research Center of Wheat and Corn Further Processing, Zhengzhou 450001, China; 3 School of Food and Biological Engineering, Luohe Food Engineering Vocational University, Luohe 462001, China; cuishengwen@sina.com

**Keywords:** genetically modified soybean, refined soybean oil, oil processing, transgenic detection, degumming, DNA fragmentation

## Abstract

Soybean is the most widely cultivated genetically modified (GM) crop worldwide, making the detection of GM soybean oil a critical issue for food safety and trade compliance. Such detection depends on whether DNA survives oil processing, yet how processing affects DNA in refined oil has remained unclear. Here, we tracked DNA mass and gene integrity along a complete industrial soybean oil processing chain, from seed to deodorized oil, and compared three degumming methods at laboratory scale. DNA mass dropped sharply during crude oil extraction and degumming, the larger part of the loss occurring between the meal and the crude oil, leaving refined oils with extremely low levels (2.60 × 10^−2^ to 7.43 × 10^−1^ ng/g). During degumming, most DNA moved from crude oil into the oil sediment, with removal efficiency decreasing in the order enzymatic > acid > hydration degumming, in line with phospholipid removal. The *lectin* and *cp4 epsps* genes degraded mainly during solvent extraction and deodorization. These findings reveal where GM DNA is lost or destroyed along the chain, show that fragments below 200 bp survive to fully refined oil, and identify enzymatic degumming as the most effective treatment for removing DNA (at the lab scale). Testing of refined soybean oil therefore requires short amplicons, and a negative result obtained with longer targets does not demonstrate the absence of GM material.

## 1. Introduction

Since being approved for commercial cultivation in 1996, genetically modified (GM) crops have expanded rapidly worldwide. In 2025, the total planting area of GM crops reached approximately 216.0 million hectares, with GM soybeans accounting for nearly 50% of this total [1]. GM soybeans are pivotal in maintaining the global supply of edible vegetable oil, supporting feed manufacturing, and sustaining the development of animal husbandry. With the worldwide adoption of GM crops, particularly the accelerated growth of soybean cultivation and international trade, the testing and regulatory oversight of GM soybeans has become an increasingly salient concern in food safety governance and trade administration. GM varieties dominate global soybean consumption. In 2025, global soybean production reached approximately 429.46 million metric tons, whereas worldwide soybean oil output was approximately 72.36 million metric tons [2]. Given an average oil content of 20% in soybeans [3], it can be inferred that approximately 80% of global soybeans are processed for oil production, making oil extraction the predominant processing pathway for GM soybeans. As the major processed derivative of GM soybeans, the detection of transgenic soybean oil is essential for enforcing labeling regulations and associated administrative measures governing GM product circulation across countries and regions.

Transgenic oilseeds and oils can be identified using multiple analytical targets, including oil content, oil quality indicators (fatty acid profile, triglycerides, and tocopherols), newly expressed proteins, and nucleic acids. Among these, DNA-based approaches are considered classical strategies for transgenic identification due to the specificity and relative stability of DNA [4]. DNA-based methods are important tools for detecting adulteration, preventing mislabeling, and ensuring the traceability of vegetable oils [5]. Notably, the quality of DNA extracts is critical for GM testing and DNA quality is influenced by DNA degradation and purity. DNA degradation can occur during food processing and storage, and the extent of degradation is closely associated with factors such as food matrix composition, elevated temperatures, and high-pressure treatments [6,7]. In addition, residual constituents, including lipids, carbohydrates, and extraction buffer salts, may inhibit PCR amplification [8]. Accordingly, most published studies have emphasized the optimization of DNA extraction methodologies [9,10,11]. Although such optimization is undeniably important, the state of DNA within food matrices is fundamentally shaped by processing operations. Nevertheless, the influences of processing parameters on DNA are frequently underestimated, even for soybean oil processing.

Degumming is the first step of oil refining, in which phospholipids and associated colloidal impurities are removed from crude oil by treatment with water, acid or phospholipase and separated as the oil sediment [12]. The removal of phospholipids is necessary, since residual phospholipids cause cloudiness and foaming, accelerate color deterioration, and reduce oxidative stability. With respect to soybean oil processing, Pauli et al. [13] reported that DNA was completely removed from degummed oil. Debode et al. [14] analyzed crude, neutralized, decolorized and deodorized soybean oils obtained from industrial facilities and demonstrated that the *lectin* gene was detectable only in crude oils by real-time PCR. Gryson et al. [15] performed lab-scale refining of crude soybean oil and observed that no DNA could be amplified in degummed oil; the same authors subsequently showed that the *lectin* gene remains detectable in degummed oil, but only at test portions of 250–365 g [16]. Collectively, these findings indicate that degumming is a critical step in DNA removal during soybean oil refining. However, by sampling a full industrial soybean oil processing chain, Costa et al. [17] showed that an 81 bp *lectin* fragment persisted through to the fully refined oil, while an 83 bp *cp4 epsps* fragment remained detectable throughout the chain except in the neutralized, washed and bleached oils, providing evidence that GM material can be traced in refined oil. Several other studies [18,19,20] have also detected the *lectin*, *cp4 epsps*, and *internal transcribed spacer* genes in refined soybean oil. The discrepancy between the two sets of conclusions may be attributed to differences in raw materials, DNA extraction protocols, DNA amplification strategies, and soybean oil processing conditions. These investigations provide valuable insights into DNA detection in individual oil samples; however, the amount of DNA remaining at each stage and the extent to which it has been degraded there have not been determined. In the studies cited above [14,15,17], the fate of DNA was assessed by endpoint or real-time PCR with targets of about 100 bp, which shows whether amplifiable DNA is present but gives no information on either the amount of DNA contained in the matrix or the lengths of the fragments that have been destroyed. Because such short targets remain amplifiable irrespective of the treatment applied, the contribution of the individual refining steps to degradation could not be separated. In addition, DNA removal has been attributed to degumming as a single operation, and the amount of DNA remaining in the oil after different degumming routes has not been compared.

Accordingly, this study aimed to systematically elucidate the distribution, persistence and degradation of soybean DNA along the oil processing chain by establishing where soybean DNA is lost along the chain and how far it is degraded at each step, determining the fragment length below which the *lectin* and *cp4 epsps* targets remain detectable in refined oil, and examining whether the degumming method influences the amount of DNA entering refining. To this end, the DNA mass concentration was quantified at each stage along two industrial processing chains, fragments of different lengths were amplified, and hydration, acid and enzymatic degumming were compared at laboratory scale. These results are expected to inform the choice of amplicon lengths for qualitative GM analysis of refined oil, the interpretation of negative results, and the regulatory assessment of processed soybean oil products.

## 2. Materials and Methods

### 2.1. Samples

**Figure 1:**
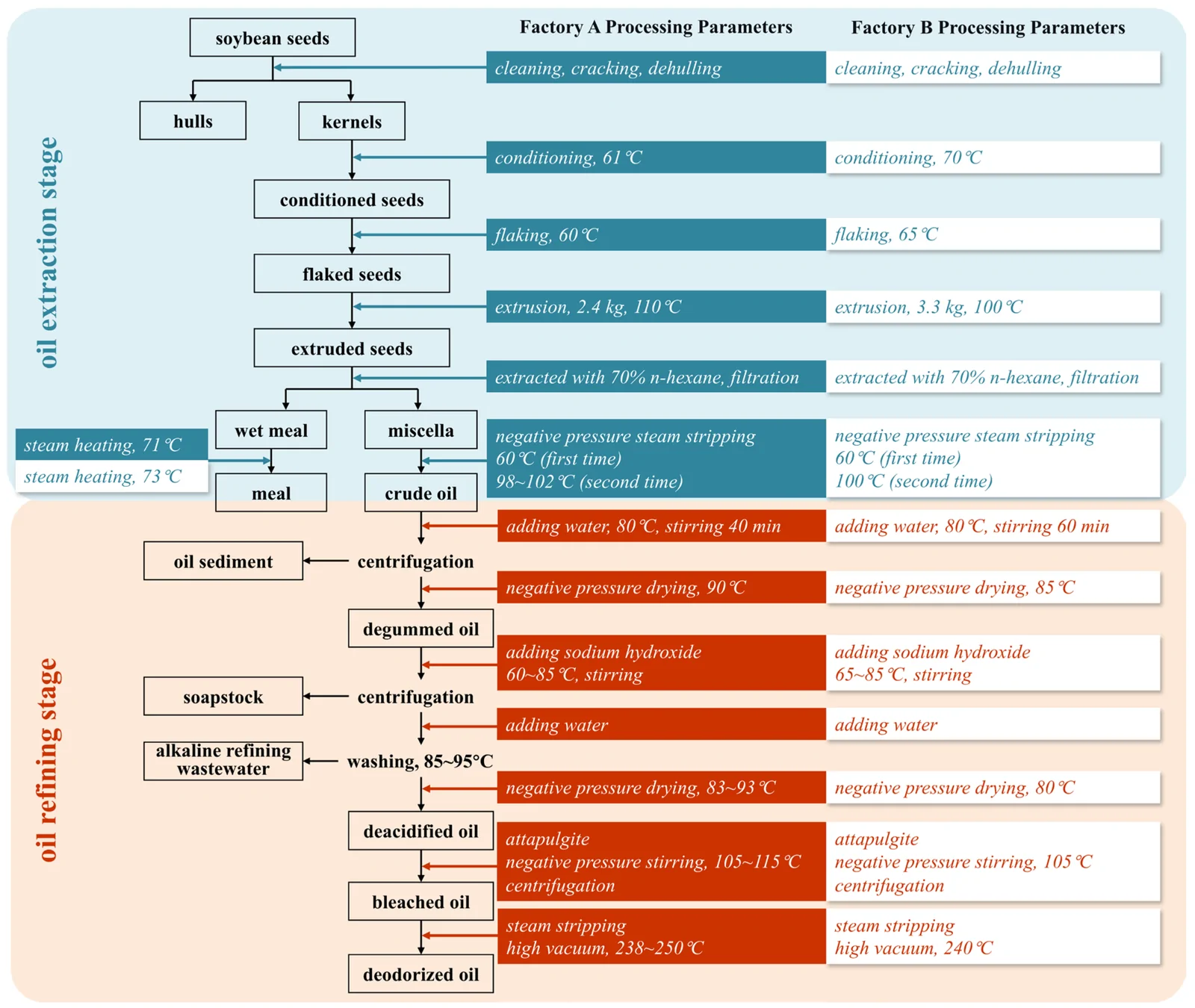
Flowchart of soybean oil processing.

All samples were collected from the production lines of two industrial soybean oil processing plants (Factory A, Lianyungang, China; Factory B, Taizhou, China). Soybeans processed at both facilities were imported from Brazil and tested positive for 5-enolpyruvylshikimate-3-phosphate synthase via immunochromatographic strip (Artron Laboratories Inc., Burnaby, BC, Canada) assays, confirming Roundup Ready-type (*cp4 epsps*-bearing) transgenic material. Soybean oil processing comprises two stages: extraction and refining, the workflow of which is illustrated in Figure 1. Samples were collected at key intermediate points, including source seeds, hulls, kernels, conditioned soybeans, flaked soybeans, extruded soybeans, meal, and crude oil from the extraction stage, as well as degummed, deacidified, bleached, and deodorized oils from the refining stage. All samples were stored at −20 °C until analysis.

Certified reference materials (CRMs) consisting of soya seed powder with different mass fractions of GTS 40-3-2 soybean (<0.09 g/kg-BF410ap, >985 g/kg-BF410bp), certified by the Joint Research Centre of the European Commission (Geel, Belgium), were purchased from Fluka (Buchs, Switzerland) and used as standards.

### 2.2. DNA Extraction

Unless otherwise stated, common chemicals and buffers used for DNA extraction were purchased from Sangon Biotech (Shanghai) Co., Ltd., Shanghai, China. The magnetic beads were purchased from BEAVER (Suzhou) Bio-medical Technology Co., Ltd., Suzhou, China.

Source seeds, hulls, kernels, conditioned soybean, flaked soybean, extruded soybean, meal, CRM-BF410ap, and CRM-BF410bp: DNA was extracted from a 100 mg aliquot of each matrix as previously described [21]. The isolated DNA was dissolved in 200 µL TE buffer (10 mM Tris, 1 mM EDTA-2Na, pH 8.0) and stored at −20 °C until use.

Crude, degummed, deacidified, bleached, and deodorized oils: DNA was extracted from 60 mL of each oil using the cetyltrimethylammonium bromide (CTAB)–magnetic bead method, as previously reported [22]. The isolated DNA was dissolved in 100 µL of TE buffer and stored at −20 °C until use.

Oil sediment: In a 50 mL tube, 25 mL of chloroform was mixed with 1–2 g of the sample and rotated at 45 rpm for 1 h using an MX-RL-Pro LCD Digital Rotator (DLAB Scientific, Beijing, China). Subsequently, 10 mL of pre-warmed (65 °C) CTAB extraction buffer was added, followed by rotation at 45 rpm for 3 h on the same instrument. After centrifugation at 12,000× *g* for 10 min at 20 °C, the upper aqueous phase from two tubes was transferred to a new 50 mL tube. DNA precipitation, purification, and washing were performed as described in the CTAB–magnetic bead method [22]. Isolated DNA from oil sediment was dissolved in 40 µL TE buffer and stored at −20 °C until use.

### 2.3. Ultraviolet Spectrophotometry

DNA yields for source seeds, hulls, kernels, conditioned soybean, flaked soybean, extruded soybean, meal, crude oil, CRM-BF410ap, and CRM-BF410bp were quantified by measuring the absorbance at 260 nm using a Nanodrop 2000 spectrophotometer (Thermo Fisher Scientific, Waltham, MA, USA). DNA purity was assessed using the absorbance ratio at 260 and 280 nm (A_260/280_ ratio).

DNA yields determined using a Nanodrop 2000 spectrophotometer were expressed as ng/μL in the TE buffer. DNA was extracted from approximately 100 mg of solid raw material and dissolved in 200 μL of TE buffer. Accordingly, the DNA concentration of solid raw material was converted from ng/μL to ng/g using the following equation:

$$C_{2}\;(\mathrm{ng}/g)=\frac{C_{1}\times 200}{m_{1}}$$

where *C*_1_ represents the DNA mass concentration (ng/μL); *m*_1_ is the mass of the solid raw materials (g); *C*_2_ denotes the DNA mass concentration (ng/g).

For crude oil, DNA was extracted from 60 mL of crude oil and dissolved in 100 μL TE buffer. Therefore, the DNA concentration was converted from ng/μL to ng/g using the following equation:

$$C_{4}\;(\mathrm{ng}/g)=\frac{C_{3}\times 100}{m_{2}}$$

where *C*_3_ represents the DNA mass concentration (ng/μL); *m*_2_ is the mass of the oil material (g); *C*_4_ denotes the DNA mass concentration (ng/g).

### 2.4. Real-Time PCR

DNA yields from crude oil, oil sediment, degummed oil, deacidified oil, bleached oil, and deodorized oil were quantified using real-time PCR.

Oligonucleotide primers targeting *lectin* (GenBank: K00821.1) and *cp4 epsps* (GenBank: AB209952.1) were used to evaluate the DNA yield. The primer pair 5′-ACGGCACCCCAAAACCCTC-3′ and 5′-GCGAAGCTGGCAACGCTAC-3′ (94 bp, Lectin-F/R1) was used in the q-Lectin amplification system, whereas 5′-CAACCACGTCTTCAAAGCAAGT-3′ and 5′-GGAAGGGTCTTGCGAAGGA-3′ (92 bp, 35S-F/R1) was used in the q-35S amplification system. Primers were synthesized by Sangon Biotech.

Each 20 μL reaction mixture contained isolated DNA (1 μL for crude oil and 5 μL for degummed oil, oil sediment, deacidified oil, bleached oil, and deodorized oil), 1× TB Green™ Premix Ex Taq™ II (TaKaRa Bio, Shiga, Japan), 1× ROX reference dye (TaKaRa Bio), and 150 nM of each primer. Amplification was performed using a Quant Studio 3 Real-Time PCR System (Applied Biosystems, Foster City, CA, USA) under the following conditions: initial pre-heating at 95 °C for 10 min, followed by 40 cycles at 95 °C for 15 s and 60 °C for 1 min, with a single fluorescence reading recorded at the end of each cycle. A no-template control using sterile water and a negative control using DNA extracts from CRM-BF410ap were included in each assay.

Two standard curves corresponding to q-Lectin and q-35S were constructed using CRM-BF410bp extracts. A 10-fold serial dilution series (510, 51, 5.1, 0.51, and 0.05 ng/μL) was prepared, and the quantification cycle (Cq) values were determined in triplicates for each dilution. Standard curves were generated by regressing the DNA yield against the corresponding Cq values [*Cq* = a × lg(*C*_DNA_) + b].

DNA yields quantified by real-time PCR were reported as ng/μL in TE buffer. DNA was extracted from 60 mL of oil and dissolved in 100 μL TE buffer. Therefore, the conversion of DNA concentration from ng/μL to ng/g followed the same equation as described for crude oil in Section 2.3.

### 2.5. Different Degumming Methods

The crude oil used for laboratory degumming was sourced from Factory B.

#### 2.5.1. Hydration Degumming

Hydration degumming was performed following the conventional lab-scale protocol [23], with minor adaptations (vessel size, water addition mode, etc.). Soybean crude oil (150 g) and a magnetic rotor were placed in a 500 mL three-necked flask and heated to 85 °C under stirring (200 r/min). Subsequently, a defined amount of water, corresponding to 3.25 times the phospholipid mass in the oil and pre-heated to 85 °C, was added dropwise, followed by stirring for 30 min (450 r/min). Thereafter, the stirring speed was reduced to 100 r/min to facilitate colloidal flocculation, and stirring was discontinued once a clear separation between the colloid and oil phases was achieved. The mixture was then centrifuged at 4500 rpm for 10 min as soon as possible. The upper oil phase was transferred to a clean 500 mL three-necked flask containing a magnetic rotor and subjected to vacuum drying by heating at 110 °C for 15 min (300 r/min) and subsequently at 120 °C for 10 min (300 r/min). The heat source was then switched off, and the vacuum was released when the oil temperature decreased to 70 °C (300 r/min). Stirring (200 r/min) was continued until the oil reached room temperature.

#### 2.5.2. Acid Degumming

Acid degumming followed the classical acid-conditioning principle [24] with minor modifications (beaker size, homogenization mode, two-step heating, etc.). Soybean crude oil (150 g) and a magnetic rotor were placed in a 500 mL high-type beaker and heated to 70 °C under stirring (200 r/min). Citric acid solution (45%, *w*/*v*; Aladdin Biochemical Technology Co., Ltd., Shanghai, China), at 0.06% of the oil volume, was added and homogenized at 10,000 rpm for 2 min. The mixture was then stirred at 70 °C for 30 min (200 r/min) and transferred to a 500 mL three-necked flask. Subsequent operations, including heating to 85 °C, water addition, stirring, centrifugation, dehydration, and cooling, were performed as described in Section 2.5.1.

#### 2.5.3. Enzymatic Degumming

Enzymatic degumming followed the established PLA1 protocol [25] with minor modifications (batch size, homogenization mode, etc.). Soybean crude oil (50 g) and a magnetic rotor were placed in a 500 mL high-type beaker and heated to 70 °C under stirring (200 r/min). Citric acid solution (45%, *w*/*v*), at 0.06% of the oil volume, was added and homogenized at 10,000 rpm for 2 min. After stirring at 70 °C for 30 min (200 r/min), sodium hydroxide solution (4%, *w*/*v*; 144 μL) was added, and the mixture was stirred at 70 °C for 5 min (200 r/min). Subsequently, a defined amount of water, corresponding to 3.25 times the phospholipid mass in the crude oil and pre-heated to 70 °C, was added dropwise, followed by homogenization at 10,000 rpm for 2 min. At this stage, the pH of the system was approximately 6.8. PLA1 (Novozymes A/S, Bagsvaerd, Denmark) was added at 40 mg enzyme/kg oil and homogenized at 10,000 r/min for 2 min. The mixture was transferred to a 250 mL flask and incubated at 50 °C for 3.5 h under stirring (300 r/min), after which it was heated at 90 °C for 10 min to inactivate the enzyme (200 r/min). The mixture was centrifuged at 10,000 *g* for 10 min as soon as possible, and the upper oil phase was collected into a 500 mL three-necked flask for vacuum drying, as described in Section 2.5.1.

### 2.6. Phosphorus Content Determination

The phosphorus content of crude soybean oil and degummed oil was determined by using the molybdenum blue colorimetric method in accordance with AOCS Official Method Ca 12–55. The phospholipids in the oil samples were first ashed in a muffle furnace ((Shanghai Jiecheng Experimental Instrument Co., Ltd., Shanghai, China), converting the phosphorus to phosphorus pentoxide. The resulting phosphorus pentoxide was then converted to phosphoric acid by boiling with hydrochloric acid (Tianjin Kemiou Chemical Reagent Co., Ltd., Tianjin, China). The phosphoric acid reacted with sodium molybdate (Aladdin) to form sodium phosphomolybdate, which was subsequently reduced to molybdenum blue with hydrazine sulfate (Aladdin). The absorbance of molybdenum blue was measured at 650 nm using a spectrophotometer (Beijing Purkinje General Instrument Co., Ltd., Beijing, China), and the phosphorus content was calculated from a standard curve.

### 2.7. Qualitative PCR

To evaluate soybean DNA degradation during soybean oil extraction, qualitative PCR was performed to amplify *lectin* and *cp4 epsps* fragments of different lengths. Primer sequences and amplification conditions are provided in Table 1. All primers were synthesized by Sangon Biotech.

Conventional PCR was conducted in 25 μL reaction volumes containing 1× Premix Taq^TM^ (Takara Bio), 0.12 μM of each primer, and 150 ng template DNA. Amplification was carried out in a TECHNE TC-412 thermal cycler (Staffordshire, UK) using an initial denaturation at 95 °C for 5 min, followed by 35 cycles of denaturation at 94 °C for 30 s, annealing for 30 s at the specified temperature (Table 1), and extension at 72 °C for varying durations (Table 1), with a final extension at 72 °C for 10 min.

### 2.8. Two-Round Nested Qualitative PCR

Because DNA concentrations in degummed, deacidified, bleached, and deodorized oils were relatively low, two-round nested qualitative PCR targeting *lectin* and *cp4 epsps* was performed to assess DNA degradation during soybean oil refinement. Primer sequences and amplification conditions are presented in Table 2. All primers were synthesized by Sangon Biotech.

The total reaction volume in both the first and second rounds of nested PCR was 25 μL, and each reaction contained 1× Premix Taq^TM^ (TaKaRa Bio). The first-round reaction mixture contained 40 nM primers and 5 μL template DNA, whereas the second-round reaction mixture contained 400 nM primers and 3 μL of the first-round PCR product. Thermal cycling was performed in a Techne TC-412 Thermal Cycler under the following program: initial denaturation at 95 °C for 5 min, followed by 35 cycles of denaturation at 94 °C for 30 s, annealing for 30 s at the specified temperature (Table 2), and elongation at 72 °C for 30 s (except Lectin-F/R3), followed by final extension at 72 °C for 10 min.

### 2.9. Agarose Gel Electrophoresis

Agarose was dissolved in 1× TAE buffer (40 mM Tris base, 20 mM acetic acid, 1 mM EDTA, pH 8.3; Sangon Biotech). After cooling to ~55 °C, 10 µL of 4S Green Plus Nucleic Acid Stain (10,000× in water; Sangon Biotech) was added to 100 mL of the molten gel solution to yield a 1× working concentration, mixed, and poured into a casting tray. After inserting the comb, the gel was allowed to solidify at room temperature for 30 min before use. Amplification products were mixed with 6× Loading Buffer (Sangon Biotech), ensuring a final 1× loading buffer concentration in the sample. A 5 µL aliquot of each mixture was loaded per well. The electrophoresis chamber was filled with 1× TAE buffer, and electrophoresis was performed at a constant 100 V for approximately 35 min. For PCR products longer than 400 bp and shorter than 400 bp, 1.5% (*w*/*v*) and 2.5% (*w*/*v*) agarose gels were used, respectively. Gel images were captured and analyzed using a UV Gel Doc 2000 system (Bio-Rad Laboratories, Hercules, CA, USA) with Quantity One software (version 4.6.2).

### 2.10. Data Analysis

For samples from the two industrial soybean oil processing plants, DNA extraction, concentration determination, qualitative PCR amplification, and nested PCR amplification were performed with three extraction replicates each. Hydration, acid, and enzymatic degumming were carried out in duplicate, with the corresponding phosphorus content derived from two extraction replicates; for DNA concentration assessment, three technical replicates (from the same DNA extract) were set per extraction replicate. One-way ANOVA was applied to assess the significant differences among independent groups. Statistical analyses were performed using SPSS software (version 21.0; IBM Analytics, Armonk, NY, USA).

## 3. Results and Discussion

### 3.1. DNA Distribution During Soybean Oil Processing

The DNA mass concentrations and purities of the key intermediate products during soybean oil processing are presented in Table 3. Soybean oil processing comprises two stages: oil extraction (stage 1) and oil refining (stage 2). Although applying a single quantification method to both processing stages is scientifically preferable, the extreme variation in DNA concentrations necessitated distinct approaches. The Nanodrop 2000 (detection limit of ~2 ng/μL) is unsuitable for stage 2 samples, as concentrations below the detection limit would falsely suggest complete DNA removal during degumming—contradicting real-time PCR evidence. Meanwhile, the dynamic range of our real-time PCR standard curve (derived from CRM-BF410bp extracts) was insufficient for the high-concentration stage 1 intermediates. Therefore, we quantified stage 1 samples using the Nanodrop 2000 spectrophotometer and stage 2 samples using real-time PCR (the mean value derived from the q-Lectin and q-35S quantitative assays). The DNA mass concentrations quantified here represent the total DNA in the matrix. Crude oil is an intermediate product shared by both stages 1 and 2. Preliminary experiments indicated that the DNA concentration in crude oil was approximately 100 ng/μL. Both the Nanodrop 2000 spectrophotometer and the real-time PCR assay were suitable for quantification, and no significant difference was observed between the two methods (for the crude oil from Factory B: 99.3 ± 2.5 ng/μL by Nanodrop 2000 vs. 93.850 ± 5.546 ng/μL by q-35S quantitative assay; *p* > 0.05).

As shown in Table 3, during soybean oil extraction (stage 1), except for soybean hulls, DNA concentrations in the source seeds, hulls, kernels, conditioned soybeans, flaked soybeans, extruded soybeans, and meal ranged from 1404.0 to 2073.7 ng/μL. The corresponding A_260/280_ ratios ranged from 1.786 to 2.000, indicating the high purity of extracted DNA. The DNA concentration in crude oil, which belonged to both stages 1 and 2 ranged from 99.3 to 120.3 ng/μL. For real-time PCR quantification, two standard curves corresponding to the q-Lectin and q-35S amplification systems were established (Table A1). The R^2^ values were 0.9918 and 0.9945, respectively, and the amplification efficiencies were 93.29% and 100.04%, demonstrating robust calibration performance suitable for subsequent quantitative analysis [29]. The DNA mass concentrations extracted from the degummed, deacidified, decolorized, and deodorized oils from both factories were all below 1 ng/μL (Table 3).

Across soybean oil processing, DNA mass concentrations in solid intermediate products remained consistently high, ranging from 10^5^ to 10^6^ ng/g. In crude oil, the DNA mass concentration decreased markedly to approximately 10^2^ ng/g, followed by a further pronounced decline in degummed, deacidified, decolorized, and deodorized oils to 10^−2^–10^−1^ ng/g (Table 3). These results indicate two major reductions in DNA mass concentration during processing: during the transition from meal to crude oil and from crude oil to degummed oil. Debode et al. [14] reported fluorometric values of 157–169 ng/mL for crude, neutralized, decolored, and refined soybean oil, corresponding to 3.925–4.225 ng/g. These values are lower than those obtained here for crude oil (165.5–200.5 ng/g) and about two orders of magnitude higher than those for refined soybean oil (0.026–0.036 ng/g). Gryson et al. [15] reported 1.86 µg/g in crude non-degummed oil, about one order of magnitude above the value obtained in this study (165.5–200.5 ng/g). The differences may be attributable to the material sampled, the DNA extraction protocol and the method of quantification. Debode et al. [14] and Gryson et al. [15] used the same physicochemical protocol, whereas this study used a physicochemical extraction combined with magnetic beads; the DNA was quantified by fluorometry in [14], by UV spectrophotometry in [15] and by real-time PCR in this study. The values reported by Debode et al. [14] were close to the limit of quantification of the fluorometric assay, which may account for the much lower concentrations obtained by these authors. The complete removal of DNA from degummed oil reported by Pauli et al. [13] and Gryson et al. [15] was limited by the sensitivity of the methods then used; the residual concentrations determined here (6.93 × 10^−1^–7.43 × 10^−1^ ng/g) indicate that degumming greatly reduces rather than eliminates the DNA content of the oil. The decrease in DNA from crude to degummed oil has been reported previously [15,16], whereas that from meal to crude oil, which accounted for the larger part of the loss, has not. Bogani et al. [30] reported lower DNA concentrations in crude oil, degummed oil and lecithin than those in seeds, crushed seeds, expander, crude flour and proteic flour, which is consistent with the present observations. Debode et al. [14] identified neutralization as the step with the strongest effect on DNA yield, whereas no significant difference was found here among degummed, deacidified, bleached and deodorized oils, most of the DNA having been removed during degumming.

The DNA extraction protocol employed in this study was specifically developed for solid soybean materials [21] and refined soybean oil [22], and demonstrates clear advantages over conventional physicochemical and commercial kit methods. These methods enable sufficient DNA recovery, thereby largely supporting the validity and scientific robustness of the conclusions. Moreover, the DNA concentrations span three to four orders of magnitude across the two steps of DNA removal: from 3.25 × 10^6^–3.38 × 10^6^ ng/g in extruded soybean to 165.5–200.5 ng/g in crude oil during extraction, and further down to 0.693–0.743 ng/g in degummed oil during degumming. Therefore, although the absence of spike-recovery correction for the DNA concentrations reported in Table 3 may partially compromise the intuitive presentation of DNA mass concentration, it does not affect the elucidation of DNA removal patterns during oil processing.

Figure 1 shows that the meal and crude oil were obtained by extracting the extruded soybeans with 70% n-hexane, followed by filtration and desolventization. Compared to extruded soybeans, the DNA mass concentration in the meal did not decrease significantly, indicating that solvent extraction did not substantially reduce DNA mass concentration. Consequently, the pronounced decrease in DNA mass concentration observed in crude oil was not attributable to the solvent extraction process itself. Given that DNA is a water-soluble and oil-insoluble molecule, the marked reduction in DNA mass concentration in crude oil was primarily associated with the intrinsic physicochemical properties of DNA.

During degumming, when the water content in the oil was low, phospholipid molecules exhibited weak polarities and high oil solubility. Upon the addition of an appropriate amount of water, phospholipid molecules undergo a structural transformation from the inner salt form to the hydrated form, thereby acquiring strong hydrophilicity [31]. As the temperature increased to the flocculation point, phospholipids absorbed water, swelled, and increased in specific gravity, leading to their precipitation from the oil phase after centrifugation. These precipitated hydrophilic phospholipids were defined as oil sediments, while the crude oil from which phospholipids had been removed was subsequently dehydrated to yield the degummed oil. Compared to degummed oil, the DNA originally present in crude oil was predominantly transferred to the oil sediment, along with hydrophilic phospholipids, resulting in a pronounced decrease in DNA mass concentration in degummed oil.

In summary, during soybean oil processing, two major decreases in DNA mass concentration occurred during the transition from the meal to crude oil and from crude oil to degummed oil. The first decrease was attributed to the preferential transfer of DNA from extruded soybeans into the meal rather than into crude oil. The second decrease resulted from the migration of DNA from crude oil into oil sediment rather than its retention in degummed oil.

### 3.2. Effects of Different Degumming Methods on DNA Removal in Crude Oil

Section 3.1 indicates that degumming is a critical step in DNA removal during the processing of soybean oil. In addition to hydration degumming, acid and enzymatic degumming are also widely applied in commercial refining and are effective approaches for degumming crude oil [32]. Accordingly, crude oil from Factory B was used as the starting material, and hydration, acid, and enzymatic degumming were performed separately on a lab-scale to evaluate their effects on DNA removal from the crude oil.

**Figure A1:**
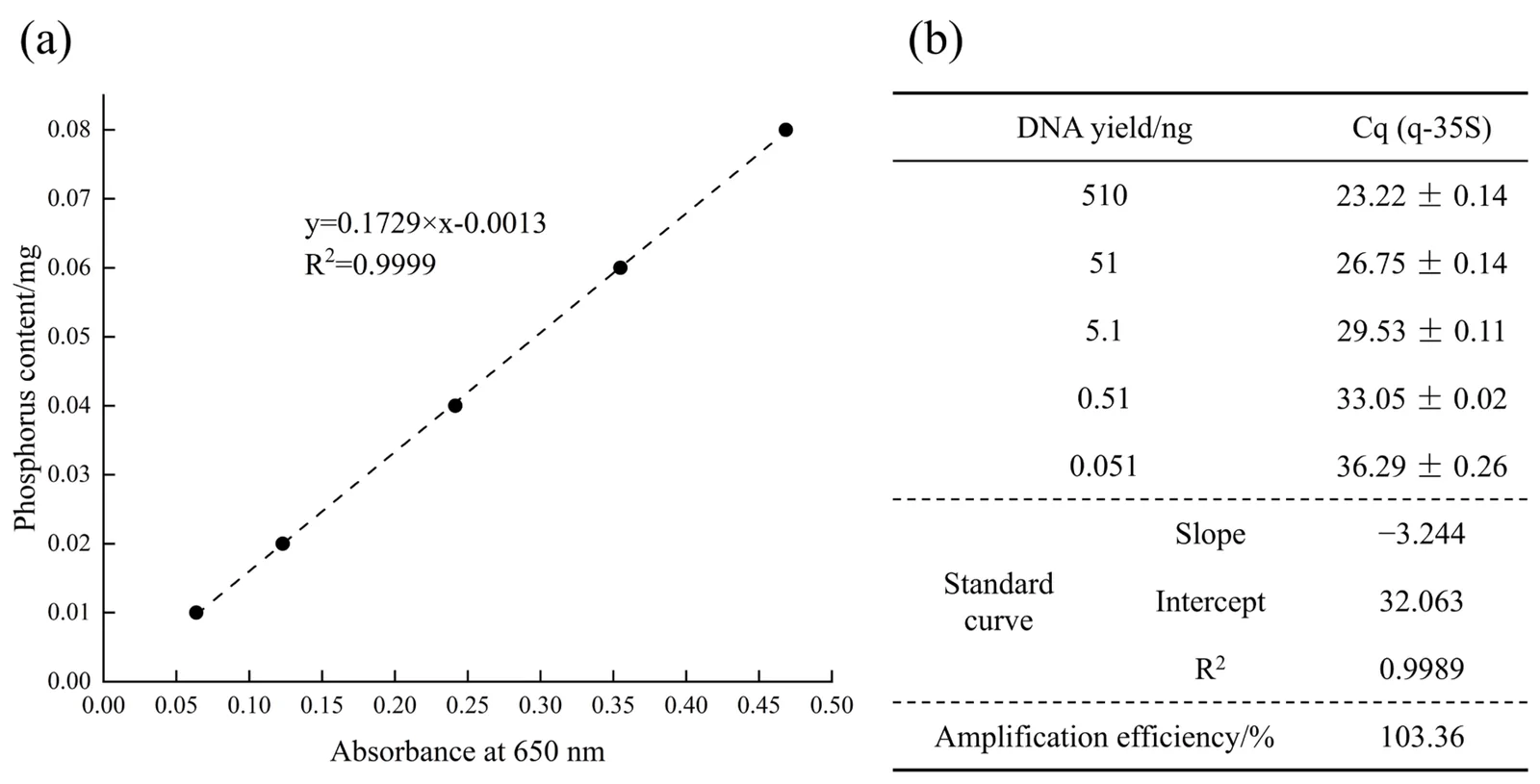
Standard curves used in Section 3.2 for (**a**) quantifying phosphorus content and (**b**) DNA mass concentration.

During crude oil degumming, the water addition level is determined by the phosphorus content of the crude oil, and the degumming performance is commonly assessed based on the residual phosphorus content in the resulting degummed oil. In the present study, the phosphorus content in the oil samples was determined using the AOCS Official Method Ca 12–55, and the corresponding calibration curve is shown in Figure A1a. The phosphorus content in degummed oil followed the order hydration degumming > acid degumming > enzymatic degumming (*p* < 0.05), whereas the phosphorus content in the corresponding oil sediment exhibited the opposite trend: hydration degumming < acid degumming < enzymatic degumming (*p* < 0.05) (Table 4). These results indicate that the phospholipid removal efficiency from crude oil ranked as follows: enzymatic degumming > acid degumming > hydration degumming. This is consistent with previous reports by Liu et al. [32] and Nikolaeva et al. [33].

The q-35S amplification system was used to quantify the DNA mass concentrations in degummed oil and oil sediment; the values represent the total DNA in the matrix (the quantitative standard curve is shown in Figure A1b). As shown in Table 5, the DNA mass concentration in the crude oil was 156.4 ng/g. Following hydration, acid, and enzymatic degumming, the DNA mass concentration in degummed oil decreased to below 1 ng/g, whereas the DNA mass concentrations in oil sediment ranged from 94.7 to 210.9 ng/g. These results indicate that, irrespective of the degumming strategy, the DNA present in crude oil is predominantly transferred to oil sediment. Compared with the DNA mass concentration of crude oil (156.4 ng/g), the DNA concentrations of oils subjected to hydration, acid, and enzymatic degumming fell within the same order of magnitude (0.360–0.652 ng/g), with corresponding Cq values ranging from approximately 33.39 to 34.22 via the q-35S amplification system (Figure A1b). A higher Cq value indicates a lower DNA mass concentration. No universal mandatory cut-off Cq value is specified in international real-time PCR guidelines for GM organism detection. Although the Cq values ranging from 33.39 to 34.22 obtained in this study are within the valid quantitative window (Figure A1b), these Cq values approach the upper limit of the conventional reliable range for precise quantification. These findings show that hydration, acid, and enzymatic degumming methods can all effectively remove exogenous genes from crude oil and the detection of GM components in degummed oil becomes considerably more challenging.

Among the three degumming methods, the DNA mass concentration in the hydrated, acid, and enzymatic degummed oils decreased sequentially, indicating that the removal efficiency of exogenous genes from crude oil followed the order enzymatic > acid > hydration degumming. As shown in Table 4, the phospholipid removal efficiency in crude oil was ranked as enzymatic method > acid method > hydrated method, which was consistent with the trend in DNA removal efficiency. This concordance can be attributed to the hydrophilic nature of both DNA and phospholipid molecules during degumming [31]; relative to the oil phase, DNA is more prone to co-migrate with phospholipids into the oil sediment. The correspondence between DNA and phospholipid removal observed here indicates that the residual DNA is carried with the phospholipids. These results provide a mechanistic basis for the relation suggested by Gryson et al. [16], in which the residual phosphorus content serves as an indicator of the residual DNA and therefore of the test portion required for a positive amplification.

As shown in Table 5, the DNA mass concentration in the oil sediment followed the order hydrated > enzymatic > acid treatment. One possible explanation is that the enhanced DNA fragmentation in acid-degummed samples could be attributed to the low pH (~3.8–4.2) introduced by citric acid conditioning, which under thermal exposure (70 °C) can accelerate acid-catalyzed depurination and subsequent strand breaks [34]. Gryson et al. [15] also noted that citric acid may impair the DNA integrity of oil sediments. However, this mechanism was not directly tested in the present study, and other processing factors (e.g., shear stress during homogenization) may also contribute. For enzymatic degumming, citric acid was initially added, then sodium hydroxide was subsequently introduced, which increased the system pH to 6.8. This condition was comparatively more favorable for DNA stability than treatment with citric acid alone. Despite the three stirring treatments, the DNA quantity in the enzymatic oil sediment remained significantly higher than that in the acidic oil sediment, indicating that stirring exerted a relatively minor influence on the DNA status in oil samples, whereas acid–base treatment had a more substantial effect.

The main conclusion of this section is that, consistent with the phospholipid removal performance in crude oil, the DNA removal efficiencies in crude oil were ranked in the order enzymatic > acid > hydration degumming. Although the removal of DNA during degumming has been reported [13,14,15,16], different degumming processes have not been compared quantitatively and enzymatic degumming remains unexamined. Variation in the degumming conditions by Gryson et al. [16] (temperature, time and the amounts of water or citric acid added) did not yield detectable DNA or amplification from the oil fractions, in contrast to the marked difference observed in this study. These results suggest that commercial oils produced by different refining processes may differ in the detectability of transgenes. Similar to Section 3.1, the DNA concentrations in Table 5 were not adjusted by spike-recovery rates, slightly limiting the interpretability of DNA partitioning during degumming. Nevertheless, the extraction protocol [22] applied to crude and degummed oils largely supports the validity of the conclusions.

It should be noted that the comparison of degumming methods presented in this Section was based on lab-scale trials. Substantial differences exist between the laboratory and industrial scales regarding the order of magnitude of crude oil. Moreover, degumming parameters may also differ between laboratory and industrial settings. For instance, following high-shear homogenization with PLA1, the reaction was stirred at 50 °C for 3.5 h in this study, whereas industrial operations typically employ a temperature range of 45–55 °C for 1–4 h [35,36]. Therefore, the removal efficiencies obtained here should be regarded as laboratory-scale values until they are confirmed under industrial refining conditions. Nevertheless, this Section characterizes the removal efficiency of exogenous genes by different degumming methods at the lab-scale, thereby providing a theoretical basis for industrial applications.

### 3.3. DNA Degradation During Soybean Oil Processing

In DNA-based transgenic detection of soybean oil, the presence and fragment length of endogenous and exogenous gene are directly associated with analytical outcomes. Therefore, the characterization of DNA degradation during soybean oil processing is essential.

As shown in Table 6, during the soybean oil extraction stage, the 1589 bp *lectin* fragment and the 2070 bp *cp4 epsps* fragment were detected in the source seeds, hulls, kernels, conditioned soybeans, flaked soybeans, and extruded soybeans. In contrast, in the meal and crude oil, the longest detectable fragments decreased to 1067 bp for the *lectin* and 1099 bp for the *cp4 epsps* genes. The targets applied to this chain previously did not exceed 1006 bp, and only those of 470 bp or less were recovered from the more highly processed materials [30]. Figure 1 illustrates that extruded soybeans were extracted with 70% n-hexane and filtered to obtain wet meal and miscella. The miscella was then subjected to primary desolventization at 60 °C and secondary desolventization at 98–102 °C to yield crude oil. These results indicate that 70% n-hexane extraction, filtration, and desolventization (60–102 °C) contributed to the detectable degradation of the 1589 bp *lectin* fragments and the 2070 bp *cp4 epsps* fragments in the crude oil. Notably, the desolventization applied to miscella constitutes a dry-heat treatment under negative pressure. As also shown in Figure 1, flaked soybeans were extruded at 100–110 °C under 2.4–3.3 kg pressure to produce extruded soybeans; however, the 1589 bp (*lectin*) and 2070 bp (*cp4 epsps*) fragments remained intact and detectable. Extrusion involves moist-heat treatment. Despite the combined application of moist heat (100–110 °C), shear, and high pressure, these longer fragments persisted. Combined treatments have been reported to induce more extensive DNA degradation than single treatments [37], suggesting that moist heat, shear, or high pressure alone is insufficient to degrade the 1589 bp (*lectin*) and 2070 bp (*cp4 epsps*) fragments. Moreover, moist-heat treatment causes more severe DNA damage than dry-heat treatment [37,38]. It can therefore be inferred that dry heat at 100–110 °C, or even at lower temperatures, is likewise incapable of causing detectable degradation of these fragments. Following the combined exposure of miscella to 70% n-hexane and desolventization (60–102 °C), degradation of the *lectin* fragments (1589 bp) and the *cp4 epsps* fragments (2070 bp) was observed in the crude oil. Therefore, the observed DNA degradation in crude oil is more likely attributable to the combined effects of organic solvent treatment and heating than to heating alone.

Because the DNA mass concentrations in degummed, deacidified, bleached, and deodorized oils were extremely low during the refining stage (Table 3), double-nested PCR was used to amplify endogenous and exogenous gene fragments. A second-round product is obtained only when the first-round target is present in the extract. Absence of the second-round product therefore indicates that the corresponding first-round fragment was either absent or present below the detection limit of the assay. As shown in Table 6, the 118 bp *lectin* and 195 bp *cp4 epsps* fragments (nested within the 438 bp and 370 bp first-round products, respectively) were detected in degummed and deacidified oils. In bleached oil, detection became intermittent: *lectin* was positive in one of three parallel reactions and *cp4 epsps* in two of three, whereas neither fragment was detected in deodorized oil. These results indicate that both target fragments were progressively degraded as refining proceeded. Using a 118 bp *lectin* target, Costa et al. [17] obtained a positive result only from crude oil and none after neutralization, whereas a 103 bp *lectin* target was amplified at every refining step including deodorization and a 106 bp *NOS 3′UTR/plant* fragment was recovered from the crude and the deodorized oils but not from the intermediate steps. The work of Bogani et al. [30] did not extend beyond the degummed oil. Deodorization was conducted at 238–250 °C under high vacuum with injected stripping steam (Figure 1), wherein thermal cleavage, limited lipid oxidation, and steam agitation coexisted. In this step, the heating represents a dry-heat treatment, with the injected steam acting as an agitation medium rather than a shear source. Notably, stripping steam agitation was also applied during conversion of miscella to crude oil. In crude oil, the 1067 bp *lectin* and 1099 bp *cp4 epsps* fragments remained detectable. Considering that combined treatments induce more extensive DNA degradation than single treatments [37], it follows that stripping steam agitation is insufficient to degrade these 1067 bp and 1099 bp fragments and, a fortiori, incapable of degrading the shorter 438 bp *lectin* and 370 bp *cp4 epsps* fragments. Furthermore, the high-vacuum environment during deodorization minimized oxidative damage to DNA. On this basis, degradation of the 438 bp *lectin* and 370 bp *cp4 epsps* fragments in deodorized oil can thus be reasonably ascribed to high-temperature dry heat (238–250 °C), rather than to oxidative damage or stripping steam agitation.

**Figure 2:**
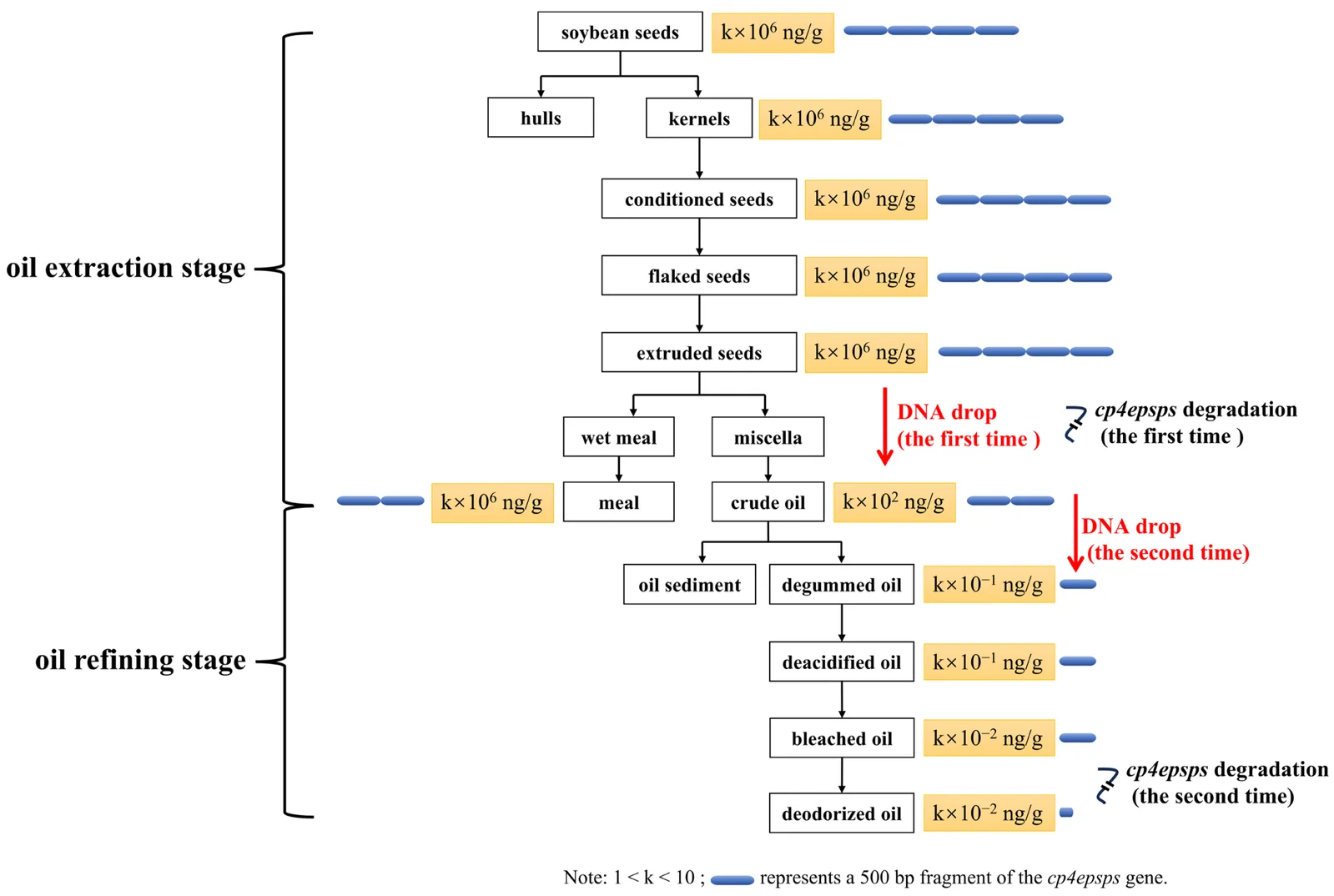
Schematic diagram of DNA distribution and *cp4 epsps* degradation during soybean oil processing.

In summary, detectable DNA degradation during soybean oil processing primarily occurred at two steps, namely extraction to obtain crude oil and deodorization to produce deodorized oil (Figure 2). Crude oil was produced from extruded soybeans using 70% n-hexane extraction, filtration, and desolventization at 60–102 °C. The degradation of endogenous and exogenous genes in crude oil results from organic solvent extraction together with heating, rather than from heating alone. In contrast, deodorized oil was produced from bleached oil via steam stripping at 238–250 °C; the degradation of endogenous and exogenous genes in deodorized oil can thus be reasonably ascribed to high-temperature heating. As also shown in Figure 2, long fragments (498 bp and 1099 bp *cp4 epsps* fragments) persist in meal and crude oil, where the DNA mass concentration is also higher, so that GM testing of these commercial materials is more readily achieved. Fully refined soybean oil, in which the DNA concentration is extremely low and the exogenous genes are extensively degraded, presents considerably greater difficulty for GM detection.

Our team previously documented the distribution and degradation of DNA during industrial soybean oil processing [27]. The sampling plants and processing parameters in the present study differ from those in the 2021 work (e.g., from flaked seeds to extruded seeds, 2.4–3.3 kg in this study vs. 4 kg in 2021; from deacidified oil to bleached oil, 105–115 °C in this study vs. 115–120 °C in 2021). Furthermore, the DNA extraction method employed herein (CTAB–magnetic bead method) contrasts with that used previously (CTAB–NucleoSpin Food Kit method); the former yields higher extraction efficiency [22] and thus more accurately reflects DNA distribution throughout refining. Against this background, the two studies converge on consistent conclusions regarding the key steps of DNA removal and the degradation of long-fragment DNA. It should be noted that data from a single plant entail limited representativeness. The two factories sampled in this study both belong to leading branded soybean oil producers in the Chinese market; together with the plant data from the preceding work [27], three plants are covered in total, thereby strengthening the representativeness of the samples. Synthesizing data from three plants, DNA removal in soybean oil processing is shown to concentrate at two stages—extraction of extruded soybean and crude oil degumming—while *cp4 epsps* gene degradation primarily occurs during extruded soybean extraction and deodorization. These outcomes partly elucidate the regularities of DNA distribution and *cp4 epsps* degradation in industrial soybean oil processing and, compared with purely laboratory-based investigations, furnish a comparatively robust reference for GM detection in commercial oils and for factory-scale transgenic load governance. Moreover, beyond reaffirming the critical role of degumming in DNA removal as reported earlier [27], the present study adds a comparative lab-scale assessment of hydration, acid, and enzymatic degumming on crude-oil DNA removal. This was designed to clarify the differential removal efficiencies under controlled conditions and thereby to guide industrial degumming selection. In summary, building upon the framework established by Xia et al. [27], the present study further validates and extends the prior conclusions on key DNA-removal steps and the degradation pattern of the *cp4 epsps* gene, through methodological renewal, expanded representativeness of industrial samples, and the inclusion of comparative degumming assays.

In deodorized oil, the 232 and 160 bp fragments of the *lectin* gene, as well as 169 and 158 bp fragments of the *cp4 epsps* gene, were successfully amplified, whereas amplification of the 438 bp *lectin* fragment and the 370 bp *cp4 epsps* fragment was unsuccessful (Table 2 and Table 6). Under the conditions of this study, shorter amplicons showed a higher probability of detection, indicating that amplicons for refined oil samples should be designed to be as short as possible. This recommendation is consistent with earlier reports on deeply processed products, which advise limiting amplicon lengths to within 300 bp [19] or 200 bp [7,39]. Chen et al. [18] went further, reporting that setting the amplicon length to 50–80 bp markedly improves the success rate of DNA-based identification and authentication of soybean oils—a range even shorter than the shortest first-round amplicon examined here (158 bp, giving a second-round product of 70 bp), further supporting the short-amplicon strategy for refined oils.

## 4. Conclusions

This study examined the complete soybean oil processing chain. The results showed that DNA was removed mainly at two stages rather than progressively: most DNA was retained in the meal after solvent extraction, only a small fraction was transferred to the crude oil (1.66 × 10^2^–2.01 × 10^2^ ng/g), and most of the DNA present in the crude oil subsequently migrated to the oil sediment during degumming, leaving only trace amounts in the degummed oil (6.93 × 10^−1^–7.43 × 10^−1^ ng/g). Thereafter, the DNA concentration remained extremely low through refining (2.60 × 10^−2^–7.43 × 10^−1^ ng/g). DNA removal efficiency corresponded to phospholipid removal efficiency, decreasing in the order enzymatic > acid > hydration degumming (lab-scale). Long fragments were degraded during solvent extraction and further degraded during deodorization, whereas fragments below 200 bp of the *cp4 epsps* gene remained detectable.

Because the data were obtained along the processing chains of two industrial plants, they are directly relevant to commercially produced oil. For refined oil, GM analysis should be performed with short amplicons (<200 bp), and failure to amplify a long target cannot be taken as evidence of the absence of GM material. For commercial meal and crude oil, in which long fragments persist and the DNA concentration is higher than in refined oil, GM testing is relatively straightforward; in fully refined oil, by contrast, the extremely low DNA concentration and the extensive degradation of the exogenous genes make detection considerably more demanding. The degumming comparison was conducted at laboratory scale, and its results remain to be validated under industrial refining conditions. These findings may provide useful references for GM detection and the regulation of soybean oil products.

## Figures and Tables

**Table 1:** Primers and amplification conditions used in qualitative PCR.

Gene	Primer	Sequence (5′-3′)	Amplicon Length (bp)	Annealing Temperature (°C)	Extension Time (s)	Reference
*lectin*	Lectin-F3	TGC CGA AGC AAC CAA ACA TGA TCC T	438	56	40	[26]
	Lectin-R3	TGA TGG ATC TGA TAG AAT TGA CGT T				
	Lectin-F4	ACC CTT GTT AGT CAA ACC ACA	1067	55	75	
	Lectin-R4	AAC CCT ATC CTC ACC CAC TCG				
	Lectin-F5	ATG GAA TAC AGC AAT GAA CA	1589	52.2	110	[27]
	Lectin-R5	GAA GGG TAG ACC AAA GAA GC				
*cp4 epsps*	EPSPS-F1	CCT TCA TGT TCG GCG GTC TCG	498	62	40	[26]
	EPSPS-R1	GCG TCA TGA TCG GCT CGA TG				
	EPSPS-F2	CGC CCG CAA ATC CTC TGG CCT TTC	1099	64	75	
	EPSPS-R2	CGT CTC GCC CTC ATC GCA ATC CAC				
	35S/NOS-F1	ATC ATT GCG ATA AAG GAA AGG C	2070	59.1	135	[27]
	35S/NOS-R1	CGT ATT AAA TGT ATA ATT GCG GGA C				

**Table 2:** Primers and amplification conditions used in two-round nested qualitative PCR.

No.	Primer	Sequence (5′-3′)	Round	Amplicon Length (bp)	Annealing Temperature (°C)	Reference
1	Lectin-F6	GCC CTC TAC TCC ACC CCC ATC C	2	118	58.0	[28]
	Lectin-R6	GCC CAT CTG CAA GCC TTT TTG TG				
	Lectin-F3	TGC CGA AGC AAC CAA ACA TGA TCC T	1	438	56.0	[26]
	Lectin-R3	TGA TGG ATC TGA TAG AAT TGA CGT T				
2	Lectin-F7	TGA CAC TAA GCC ACA AAC AC	2	86	51.5	[27]
	Lectin-R7	CAA ACT CAA CAG CGA CGA C				
	Lectin-F8	CAA CTT CAC CTT CTA TGC C	1	232	53.4	
	Lectin-R8	CGT TTT GAT GGA TCT GAT AG				
3	Lectin-F1	ACG GCA CCC CAA AAC CCT C	2	94	58.0	[27]
	Lectin-R1	GCG AAG CTG GCA ACG CTA C				
	Lectin-F2	CCT CGG GAA AGT TAC AA	1	160	54.5	
	Lectin-R2	GGG GCA TAG AAG GTG AA				
4	35S-F2	GCT CCT ACA AAT GCC ATC A	2	195	55.4	[27]
	35S-R2	GAT AGT GGG ATT GTG CGT CA				
	ESG/35S-F1	TTC AAA CCC TTC AAT TTA ACC GAT	1	370	53.6	
	ESG/35S-R1	AAG GAT AGT GGG ATT GTG CGT C				
5	35S/CTP-F1	GAC ACG CTG ACA AGC TGA CTC	2	99	50.1	[27]
	35S/CTP-R1	GGA AAT TGG AAT TGG GAT TAA				
	35S/CTP-F2	ATC CCA CTA TCC TTC GCA AGA	1	169	52.8	
	35S/CTP-R2	TGG GGT TTA TGG AAA TTG GAA				
6	EPSPS-F3	ATA TCC GAT TCT CGC TGT CGC	2	70	56.2	[27]
	EPSPS-R3	GAG TTC TTC CAG ACC GTT CAT				
	EPSPS-F4	TCG CTC CTC CAC GCT GAA	1	158	60.4	
	EPSPS-R4	AGA GGC GGT CGC TTT CC				

Note: ESG refers to the endogenous soybean gene.

**Table 3:** DNA mass concentrations of intermediate products from the soybean oil processing.

Samples	Factory A			Factory B		
	DNA Mass Concentration/(ng/μL)	A_260/280_	DNA Mass Concentration/(ng/g)	DNA Mass Concentration/(ng/μL)	A_260/280_	DNA Mass Concentration/(ng/g)
Soybean seed	1781.3 ± 30.6 ^a^	1.824–1.910	3,562,667 ^a^	1636.0 ± 224.7 ^BC^	1.809–1.840	3,272,000 ^B^
hulls	359.3 ± 51.9 ^b^	1.860–1.983	718,667 ^b^	603.0 ± 18.5 ^D^	1.981–2.000	1,206,000 ^D^
kernels	1854.3 ± 19.4 ^a^	1.786–1.819	3,708,667 ^a^	1485.0 ± 112.2 ^C^	1.806–1.857	2,970,000 ^BC^
conditioned soybean	1652.7 ± 110.2 ^a^	1.832–1.903	3,305,333 ^a^	1404.0 ± 71.8 ^C^	1.845–1.973	2,808,000 ^C^
flaked soybean	1932.3 ± 134.6 ^a^	1.816–1.862	3,864,667 ^a^	1924.0 ± 136.9 ^AB^	1.796–1.886	3,848,000 ^A^
extruded soybean	1690.0 ± 336.1 ^a^	1.809–1.926	3,380,000 ^a^	1623.7 ± 16.4 ^C^	1.844–1.970	3,247,333 ^B^
meal	1929.0 ± 201.0 ^a^	1.838–1.897	3,858,000 ^a^	2073.7 ± 159.1 ^A^	1.847–1.917	4,147,333 ^A^
crude oil	120.3 ± 17.9 ^c^	1.816–1.915	200.5 ^c^	99.3 ± 2.5 ^E^	1.817–1.824	165.5 ^E^
degummed oil	0.446 ± 0.039 ^d^	/	0.743 ^d^	0.416 ± 0.031 ^F^	/	0.693 ^F^
deacidified oil	0.128 ± 0.008 ^d^	/	0.213 ^d^	0.177 ± 0.034 ^F^	/	0.296 ^F^
bleached oil	0.039 ± 0.002 ^d^	/	0.066 ^d^	0.043 ± 0.003 ^F^	/	0.071 ^F^
deodorized oil	0.021 ± 0.010 ^d^	/	0.036 ^d^	0.015 ± 0.003 ^F^	/	0.026 ^F^

Note: DNA concentrations in crude oil were measured with a Nanodrop 2000 spectrophotometer. Different superscript lowercase or uppercase letters within a column denote significant variation (*p* < 0.05).

**Table 4:** Efficiency of different degumming methods.

Samples	Phosphorus Content/(mg/kg)		
	Hydration Degumming	Acid Degumming	Enzymatic Degumming
Crude oil	610.27 ± 17.17		
Degummed oil	128.45 ± 6.17 ^a^	4.94 ± 0.14 ^b^	0.71 ± 0.052 ^c^
Oil sediment	1755.21 ± 121.01 ^c^	2050.64 ± 121.70 ^b^	2769.20 ± 168.56 ^a^

Note: Different superscript lowercase letters within a row denote significant variation (*p* < 0.05).

**Table 5:** The efficiency of DNA removal from crude oil of different degumming methods.

Samples	DNA Mass Concentration/(ng/μL)			DNA Mass Concentration/(ng/g)		
	Hydration Degumming	Acid Degumming	Enzymatic Degumming	Hydration Degumming	Acid Degumming	Enzymatic Degumming
Crude oil	93.850 ± 5.546			156.417 ^A^		
Degummed oil	0.391 ± 0.005	0.310 ± 0.021	0.216 ± 0.033	0.652 ^Ba^	0.517 ^Cb^	0.360 ^Bc^
Oil sediment	6.855 ± 0.955	3.079 ± 0.363	5.061 ± 0.532	210.935 ^Ca^	94.746 ^Bb^	155.727 ^Aab^

Note: DNA concentration in crude oil was determined by real-time PCR assay. Different superscript uppercase letters within a column denote significant variation (*p* < 0.05). Different superscript lowercase letters within a row denote significant variation (*p* < 0.05).

**Table 6:** Amplicon sizes of *lectin* and *cp4 epsps* fragments during soybean oil processing.

Samples	*lectin* (Full-Length 2152 bp)						*cp4 epsps* (Full-Length 2457 bp)					
	Factory A			Factory B			Factory A			Factory B		
	438 bp	1067 bp	1589 bp	438 bp	1067 bp	1589 bp	498 bp	1099 bp	2070 bp	498 bp	1099 bp	2070 bp
Soybean seeds	3/3	3/3	3/3	3/3	3/3	3/3	3/3	3/3	3/3	3/3	3/3	3/3
hulls	3/3	3/3	3/3	3/3	3/3	3/3	3/3	3/3	3/3	3/3	3/3	3/3
kernels	3/3	3/3	3/3	3/3	3/3	3/3	3/3	3/3	3/3	3/3	3/3	3/3
conditioned soybean	3/3	3/3	3/3	3/3	3/3	3/3	3/3	3/3	3/3	3/3	3/3	3/3
flaked soybean	3/3	3/3	3/3	3/3	3/3	3/3	3/3	3/3	3/3	3/3	3/3	3/3
extruded soybean	3/3	3/3	3/3	3/3	3/3	3/3	3/3	3/3	3/3	3/3	3/3	3/3
meal	3/3	3/3	0/3	3/3	3/3	0/3	3/3	3/3	0/3	3/3	3/3	0/3
crude oil	3/3	3/3	0/3	3/3	3/3	0/3	3/3	3/3	0/3	3/3	3/3	0/3
	Factory A			Factory B			Factory A			Factory B		
	160 → 94 bp	232 → 86 bp	438 → 118 bp	160 → 94 bp	232 → 86 bp	438 → 118 bp	158 → 70 bp	169 → 99 bp	370 → 195 bp	158 → 70 bp	169 → 99 bp	370 → 195 bp
degummed oil	3/3	2/3	3/3	3/3	2/3	3/3	3/3	3/3	3/3	3/3	2/3	3/3
deacidified oil	3/3	2/3	2/3	2/3	3/3	3/3	3/3	2/3	3/3	3/3	3/3	3/3
bleached oil	2/3	3/3	1/3	2/3	2/3	1/3	3/3	1/3	2/3	2/3	1/3	2/3
deodorized oil	2/3	2/3	0/3	2/3	2/3	0/3	3/3	0/3	0/3	3/3	0/3	0/3

Note: The agarose gel electrophoresis results corresponding to this table are presented in Figure A2 and Figure A3. In the ratios 0/3, 1/3, 2/3, and 3/3, the denominator represents three independent extraction–amplification replicates, and the numerator indicates the count of positive amplifications; Taking 160 → 94 bp as an example: 160 bp is the first-round product, and 94 bp is the second-round product of the nested PCR.

**Figure A2:**
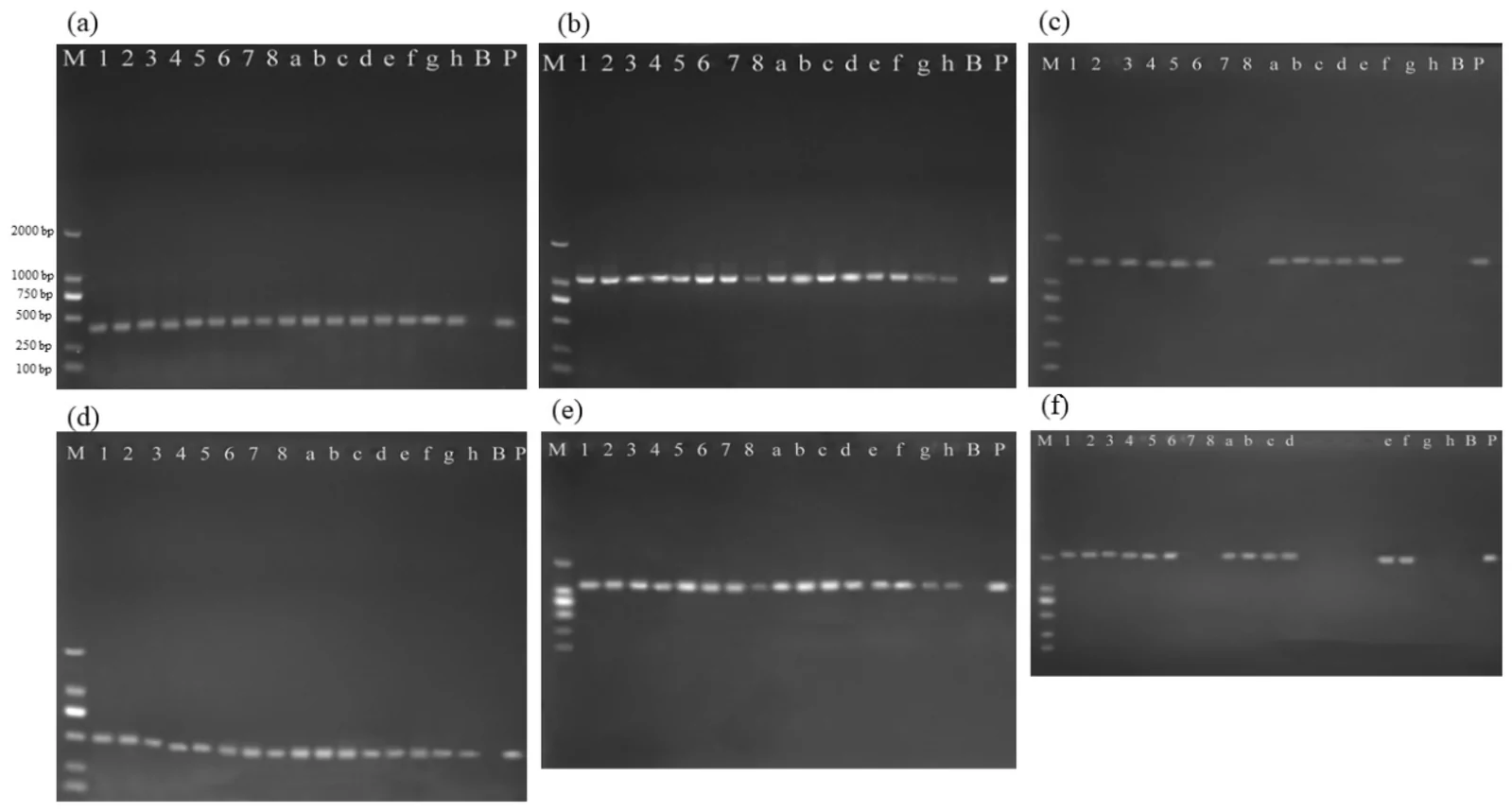
Agarose gel electrophoresis results (one replicate) of conventional qualitative PCR in intermediate products from oil extraction process. Notes: (**a**) 438 Bp *lectin*, (**b**) 1067 bp *lectin*, (**c**) 1589 bp *lectin*, (**d**) 498 bp *cp4 epsps*, (**e**) 1099 bp *cp4 epsps*, (**f**) 2070 bp *cp4 epsps*. Line M, DL2000 DNA marker (TaKaRa Bio, Shiga, Japan); Line B, blank; Line P, positive control. Line 1–8: source seeds, hulls, kernels, conditioned soybean, flaked soybean, extruded soybean, meal, and crude oil from factory A. Line a–h: source seeds, hulls, kernels, conditioned soybean, flaked soybean, extruded soybean, meal, and crude oil from factory B.

**Figure A3:**
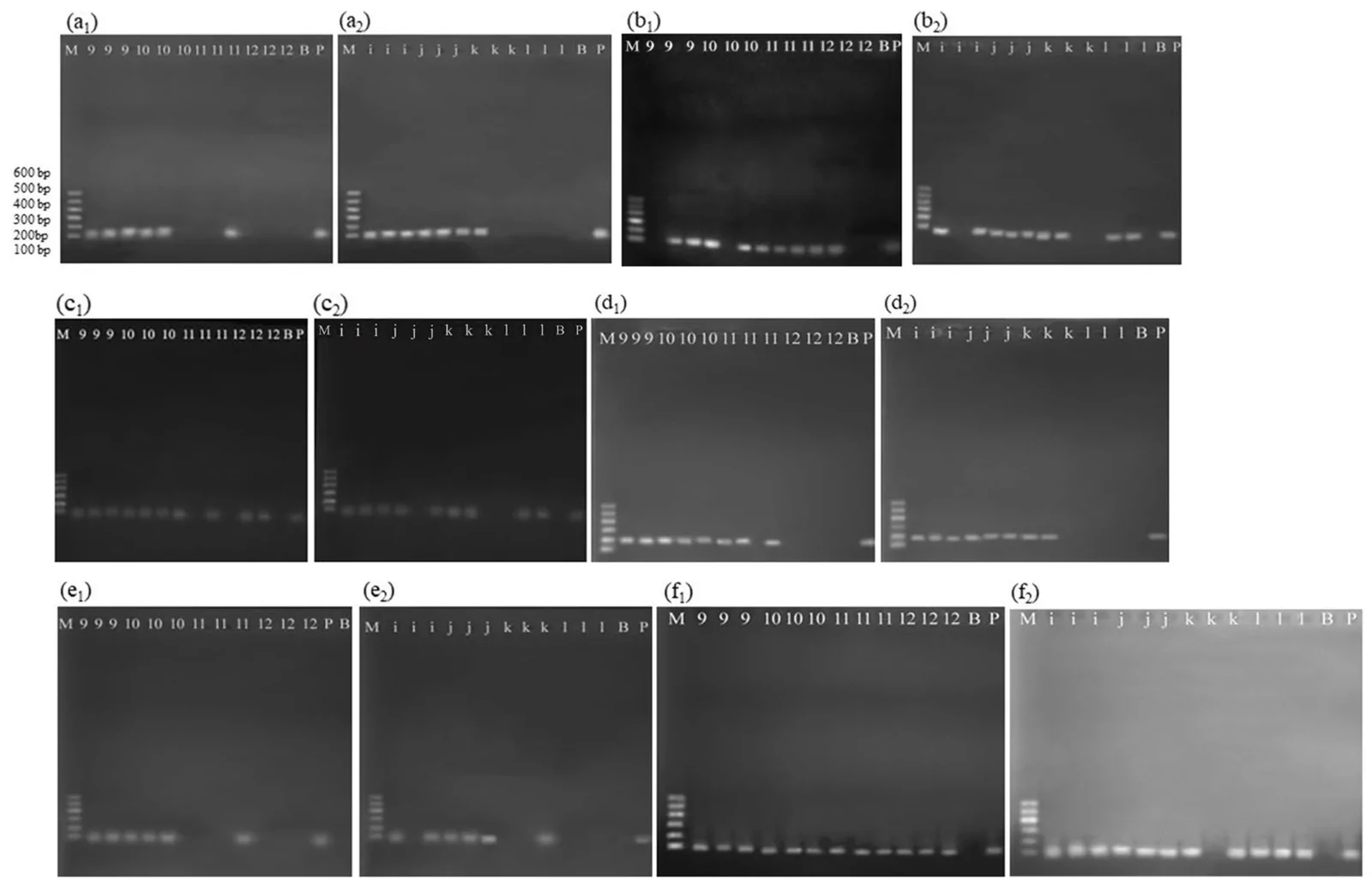
Agarose gel electrophoresis results of nested PCR in intermediate products from oil refining process. Notes: (**a_1_**,**a_2_**) 118 bp *lectin*, (**b_1_**,**b_2_**) 86 bp *lectin*, (**c_1_**,**c_2_**) 94 bp *lectin*, (**d_1_**,**d_2_**) 195 bp *cp4 epsps*, (**e_1_**,**e_2_**) 99 bp *cp4 epsps*, (**f_1_**,**f_2_**) 70 bp *cp4 epsps*. Line M, Marker I (TaKaRa Bio, Shiga, Japan); Line B, blank; Line P, positive control. Line 9–12: degummed oil, deacidified oil, bleached oil, and deodorized oil from factory A. Line i–l: degummed oil, deacidified oil, bleached oil, and deodorized oil from factory B.

## Data Availability

The original contributions presented in this study are included in the article. Further inquiries can be directed to the first author.

## Appendix A

**Table A1:** Standard curves used in Section 3.1 for quantifying DNA mass concentration of degummed oil, deacidified oil, bleached oil, and deodorized oil.

DNA Yield/ng		Cq	
		q-Lectin	q-35S
510		21.99 ± 0.07	22.35 ± 0.09
51		24.59 ± 0.14	25.05 ± 0.13
5.1		27.85 ± 0.12	28.99 ± 0.05
0.51		31.73 ± 0.23	31.56 ± 0.26
0.051		35.89 ± 0.33	35.70 ± 0.37
Standard curve	Slope	−3.494	−3.321
	Intercept	30.88	31.08
	R^2^	0.9918	0.9945
Amplification efficiency/%		93.29	100.04
